# n-Type conducting P doped ZnO thin films *via* chemical vapor deposition†

**DOI:** 10.1039/d0ra05667g

**Published:** 2020-09-17

**Authors:** Donglei Zhao, Jianwei Li, Sanjayan Sathasivam, Claire J. Carmalt

**Affiliations:** Materials Chemistry Centre, Department of Chemistry, University College London 20 Gordon Street London WC1H 0AJ UK c.j.carmalt@ucl.ac.uk

## Abstract

Extrinsically doped ZnO thin films are of interest due to their high electrical conductivity and transparency to visible light. In this study, P doped ZnO thin films were grown on glass substrates *via* aerosol assisted chemical vapour deposition. The results show that P is a successful dopant for ZnO in the V+ oxidation state and is able to reduce resistivity to 6.0 × 10^−3^ Ω cm while maintaining visible light transmittance at ∼75%. The thins films were characterized by X-ray diffraction studies that showed only Bragg peaks for the wurtzite ZnO phase. Fitting of the diffraction data to a Le Bail model also showed a general expansion of the ZnO unit cell upon doping due to the substitution of Zn^2+^ ions with the larger P^5+^.

## Abstract 2

P doped ZnO thin films showing enhanced electrical conductivity.

## Graphical abstract

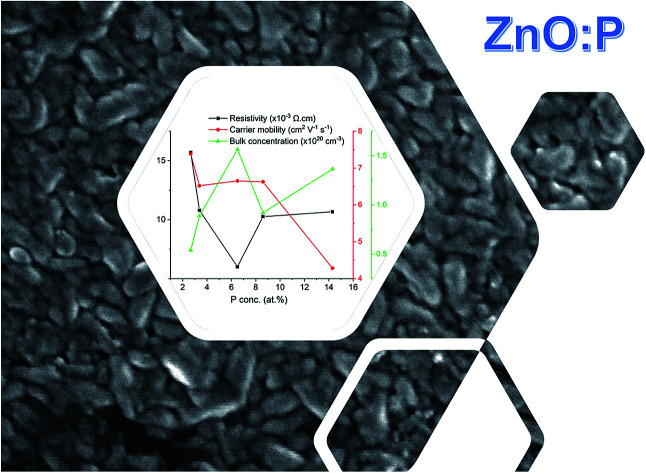

## Introduction

Transparent conducting oxides (TCOs) are important materials that are widely used in optoelectronic devices such as solar cells, touchscreens, screen displays, LCD panels and OLEDs.^1–4^ TCOs combine the seemingly orthogonal properties of high transmittance to the visible wavelengths (>80%) with low electrical resistivity (<10^−3^ Ω cm). This is achieved due to TCO materials being wide band gap (>3.1 eV) semiconductors and carrier concentrations in the 10^20^ cm^−3^ order or above due to intrinsic and/or extrinsic point defects.^1,5–8^

Currently, the most widely used TCO material is tin doped indium oxide (ITO), for example, 90% of the global display market is based on ITO transparent electrodes.^2,3,7,9,10^ ITO achieved this dominance due to its superior properties including transparencies as high as 90% and resistivities as low as ×10^−5^ Ω cm. However, the use of ITO is not sustainable owing to its high cost which is associated to supply issues of indium.^8,11–13^ As such, other semiconductor materials like SnO_2_ and ZnO have been investigated as potential replacements.

ZnO is a mechanically, optically and electrically stable and inexpensive semiconductor with a direct optical band gap of 3.37 eV (*via* spectroscopic ellipsometry)^14^ or 3.27 eV (by applying the Tauc method to UV-visible spectroscopy data).^15–21^ The intrinsic n-type conductivity in ZnO is believed to arise from adventitious H (or C), not from defects such as O vacancies (deep donors) or Zn interstitials (unstable at room temperature) as previously thought.^22^ In the nominally undoped form, ZnO is too electrically resistive for TCO applications and is therefore typically doped with trivalent donor dopants such as Al^3+^, Ga^3+^ and In^3+^ on Zn^2+^ sites.^5,23^ With these aforementioned dopants, shallow donor levels are formed that enable high electron densities (×10^20^ cm^−3^) and hence resistivities as low as 2 × 10^−4^ Ω cm.^1,15,16^ Although ZnO shows resistance in forming shallow acceptor levels allowing for p-type conductivity, as evidenced from computational and experimental studies,^24–26^ there have been numerous successful studies on p-type ZnO achieved from nitrogen (N_O_), arsenic (As_O_), phosphorus (P_O_) and lithium (Li_Zn_) but often with limited stability and requiring suppression of compensating donor states.^24–29^

Phosphorus is an interesting dopant candidate for ZnO as its multivalent nature existing in the III−, III+ and V+ states, technically allows it to be an n-type and/or p-type dopant *via* P^3+^/P^5+^ on Zn^2+^ sites or P^3−^ on O^2−^ sites, respectively. Along with p-type conductivity, previous studies have shown an enhancement in electron density and n-type conductivity originating through the formation of P_Zn_ defects and P^3+^, P^5+^ or P^3−^ related complexes.^24,30–34^

In this study, the effect of P doping on the material and optoelectronic properties of ZnO is investigated. The films were grown using AACVD a scalable, highly tunable and industrially friendly route to determine the suitability of P as a dopant for real world TCO applications and to study whether p or n type conductivity is achieved.

## Experimental

### Film synthesis

Depositions were carried out under a N_2_ (BOC Ltd., 99.99% purity) flow. Zinc acetate dihydrate (Zn(OAc)_2_·2H_2_O), triethyl phosphate ([PO(OEt)_3_] (99%) and methanol (99%)) were purchased from Merck. Films were grown on barrier coated (∼50 nm SiO_2_) float glass (5 cm × 15 cm × 0.4 cm) which were cleaned using detergent, water and isopropanol then dried in a 70 °C oven prior to deposition.

Zn(OAc)_2_·2H_2_O (0.40 g, 1.82 mmol) in methanol (20 mL) was placed in a glass bubbler. [PO(OEt)_3_] (*x* mol% based on Zn, *x* = 0, 0.5, 1.0, 5.0, 7.0 and 10.0) was added in the same bubbler. The solution was atomised through a piezoelectric device (Johnson Matthey Liquifog®). The aerosol mist was delivered to the AACVD reaction chamber and passed over the heated substrate using N_2_ carrier gas at 1.0 L min^−1^.^35^ Depositions were carried out at 500 °C and lasted until the precursor solution was fully used. After the deposition the substrates were cooled under a flow of N_2_. The glass substrate was not removed until the graphite block was cooled to below 50 °C. The films on the substrates were handled and stored in air.

### Film characterisation

The X-ray diffraction (XRD) analysis scanning from 10 to 65° (2*θ*) used a modified Bruker-AXS D8 diffractometer with parallel beam optics and a PSD LynxEye silicon strip detector. The scans used a monochromated Cu Kα source operated at 40 kV and its emission current was 30 mA with 0.5° as incident beam angle and 0.05° at 1 s/step as step frequency. X-ray photoelectron spectroscopy (XPS) analysis was used to determine the surface elemental surroundings *via* a Thermo Scientific K-alpha photoelectron spectrometer using monochromatic Al Kα radiation. Higher resolution scans were recorded for the principal peaks of zinc (Zn 2p), phosphorus (P 2p), oxygen (O 1s) and carbon (C 1s) at a pass energy of 50 eV, and then the CasaXPS software was used to analyse the data from the XPS. The binding energy of adventitious carbon was adjusted at 284.5 eV as calibration. The JEOL JSM-6301F Field Emission Scanning Electron Microscopy (SEM) with 1.5 keV as accelerating voltage was used to investigate the surface morphologies of the thin films. To avoid charging, all the samples were coated with gold before the analysis. The optical properties were determined through a Shimadzu UV-2600 spectrometer scanning between 1100 and 300 nm. Hall effect measurements were used to calculate the electrical properties including bulk concentration (*n*), carrier mobility (*μ*) and resistivity (*ρ*) *via* the van der Pauw method with a permanent magnet (0.58 T) and a constant current (1 mA).

## Results and discussion

Nominally undoped and P doped ZnO thin films on glass substrates were prepared from the AACVD reaction of zinc acetate dehydrate [Zn(OAc)_2_·2H_2_O], methanol and triethylphosphate ([PO(OEt)_3_]) at 500 °C. All ZnO films were well adhered to the substrate, passing the Scotch tape test.^36^ The bulk concentrations of P in the doped ZnO films were determined through energy dispersive X-ray spectroscopy (EDS) to be 0, 2.7, 3.4, 6.5, 8.6 and 14.3 at% for the 0, 0.5, 1, 5, 7 and 10 mol% of [PO(OEt)_3_] – respectively, relative to [Zn(OAc)_2_·2H_2_O] in the precursor solution.

**Fig. 1:**
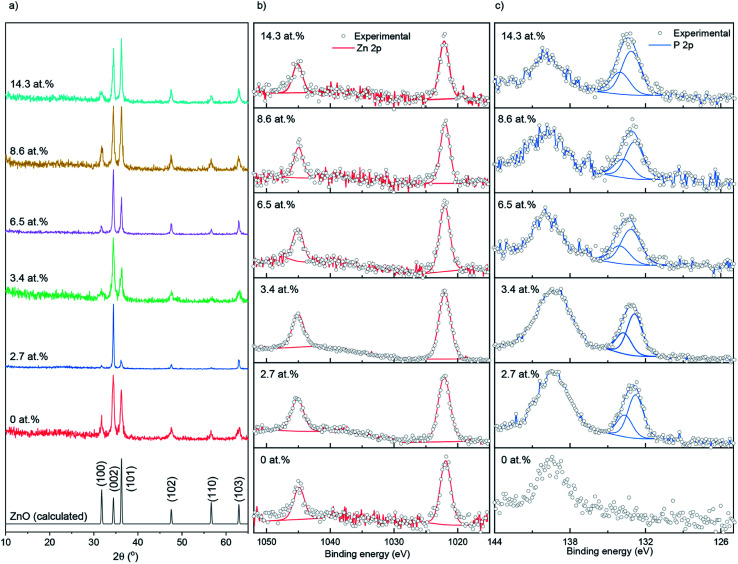
(a) XRD patterns, (b) XPS Zn 2p spectra and (c) XPS P 2p spectra for the undoped and P doped ZnO films prepared at 500 °C on glass substrates through AACVD.

The X-ray diffraction patterns of undoped and P doped ZnO films are shown in Fig. 1a. Peaks at 31.8, 34.4, 36.3, 47.5, 56.6 and 63.0° respectively correspond to the (100), (002), (101), (102), (110) and (103) planes of the wurtzite phase of ZnO. No additional peaks for secondary oxide phases such as Zn_3_P_2_ or P_2_O_5_ were visible. Calculations performed on the XRD data showed that P had little influence on the preferred orientation, with the (102) plane having the strongest preference followed by (002) and a lack of preference for the (100). This is most likely due to a combination of substrate influence and the fact that the (002) is the lowest energy surface in the wurtzite crystal structure, therefore disproportionately favored for growth.^37^ The XRD data was also used to determine the ZnO lattice parameters prior to and after P doping, the results of which are shown in Table 1, and suggest a general contraction in the hexagonal wurtzite unit cell upon P incorporation due to the smaller P^3+^ (0.44–0.58 Å) or P^5+^ (0.31–0.34 Å) occupying Zn^2+^ (0.74 Å) sites in the lattice. If P was in the III− state (1.8–2.1 Å) on O^2−^ (1.3–1.4 Å) sites, then an expansion in the ZnO unit cell would have been observed.^38^

**Table 1:** Shows the concentration of P in the AACVD grown ZnO films as well as their unit cell parameters

P conc./at%	Unit cell parameters		
	*a*/Å	*c*/Å	Volume/Å^3^
0	3.252(3)	5.211(3)	47.71
2.7	3.253(2)	5.202(0)	47.68
3.4	3.243(4)	5.206(2)	47.42
6.5	3.248(2)	5.205(1)	47.57
8.6	3.250(2)	5.210(2)	47.64
14.3	3.251(2)	5.201(2)	47.62

X-ray photoelectron spectroscopy (XPS) was used to determine the surface oxidation state and composition of the thin films (Fig. 1b and c). The Zn 2p data were fit with a doublet of peaks separated by 23.1 eV. The Zn 2p_3/2_ peaks for the doped and undoped films were centered at ∼1022.1 eV, corresponding to Zn^2+^.^39^ The P 2p experimental data were also fit with a doublet of peaks at a separation of 0.87 eV. The P 2p_3/2_ transitions were at ∼132.9 eV, corresponding to literature reports for P^5+^.^40–42^ No indication of P^3+^ or P^3−^ was found on the surface of the thin films. The peaks at ∼139 eV that were seen in the P 2p scan range belongs to Zn 3s transitions.^41^ XPS depth profiling carried out on the 14.3 at% doped film (see ESI Fig. S1†) showed the P to be surface segregated as opposed to evenly distributing across the depth of the films. Surface segregation of dopants in ZnO is common and has been seen previously in literature with anionic and cationic dopants.^43,44^

**Fig. 2:**
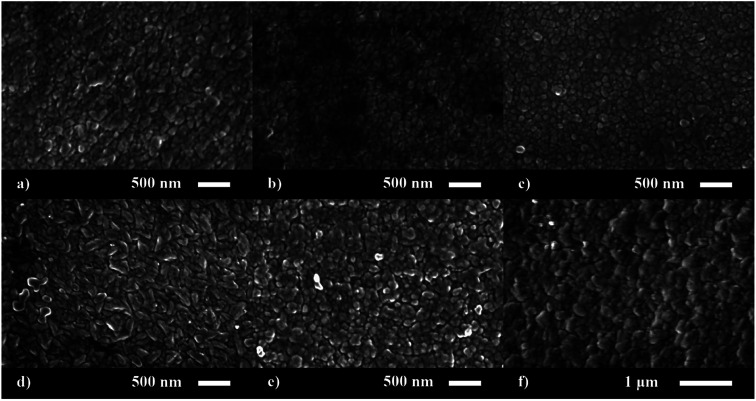
SEM images for the morphology of the (a) 0, (b) 2.7, (c) 3.4, (d) 6.5, (e) 8.6 and (f) 14.3 at% P doped ZnO films prepared *via* AACVD.

The surface morphology of the nominally undoped and series of P doped ZnO thin films were investigated *via* scanning electron microscopy (SEM) (Fig. 2). The morphology was similar to what has previously been seen for ZnO films grown from [Zn(OAc)_2_·2H_2_O] in methanol and consisted of compact dome/platelet like features. The size of the features were ∼200 nm at up to 3.4 at% doping but increased to 300–400 nm at higher concentrations of P. Side on micrographs revealed the film thickness to be 730, 850, 630, 600, 660 and 780 nm for the 0, 2.7, 3.4, 6.5, 8.6 and 14.3 at% P doped ZnO films, respectively.

**Fig. 3:**
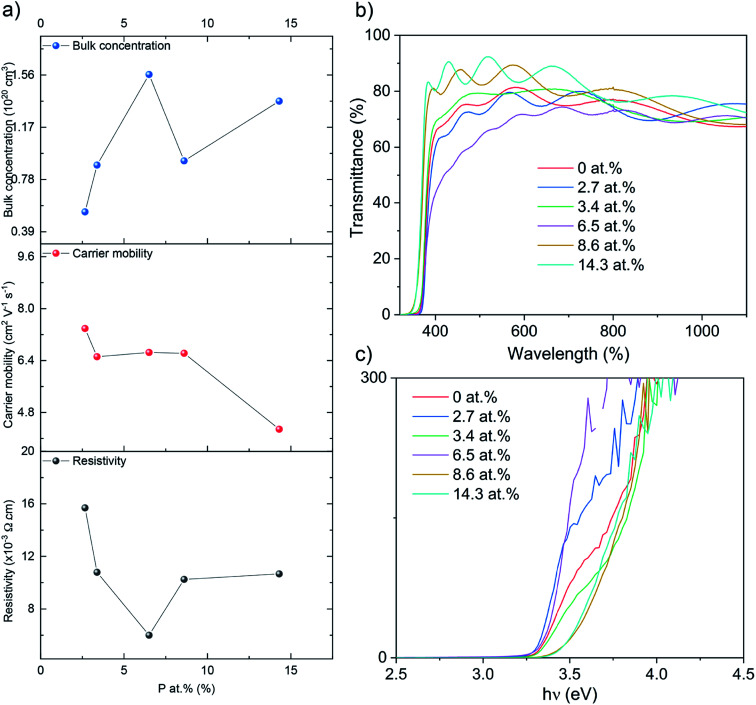
(a) The Hall data, (b) the optical data showing transmittance and (c) the Tauc plots for the nominally undoped and P doped ZnO films on glass substrates.

The electrical properties were probed *via* Hall effect measurements (Fig. 3a). The nominally undoped ZnO film was too resistive to measure *via* the Hall instrument but crude measurements of resistance using a two-point probe showed values in the MΩ region. Upon doping of ZnO with P an enhancement in the film conductivity was instantly observed to measurable levels with a negative Hall coefficient, indicative of n-type conductivity. The 2.7 at% doped film showed resistivity of 1.6 × 10^−2^ Ω cm, carrier concentration of 5.3 × 10^19^ cm^−3^ and electron mobility of 7.4 cm^2^ V^−1^ s^−1^. At 3.4 at% P, resistivity was further reduced to 1.0 × 10^−2^ Ω cm and at 6.5 at% P, the lowest resistivity of 6.0 × 10^−3^ Ω cm was achieved owing to an increase in the electron concentration of 8.9 × 10^19^ cm^−3^ and 1.6 × 10^20^ cm^−3^, respectively. The increase in the electron concentration was observed due to the successful replacement of Zn^2+^ with P^5+^, thereby releasing up to three electrons for conduction for every Zn^2+^ replaced. Further increase in P to 8.6 and 14.3 at% resulted in an increase in resistivity to 1.0 × 10^−2^ and 1.1 × 10^−2^ Ω cm due to both a decrease in carrier concentration (to 9.1 × 10^19^ and 1.4 × 10^20^ cm^−3^, respectively) and carrier mobility (6.6 and 4.3 cm^2^ V^−1^ s^−1^). The decrease in carrier concentration is attributed to self-compensating mechanisms, such as O interstitials or Zn vacancies that increase with increasing P levels in ZnO, and/or the formation of electrically inactive secondary phases such as Zn_3_P_2_ or P_2_O_5_ that were undetected by XRD and XPS.^45–47^ The reduction in carrier mobility with increasing levels of P is due to increased ionized impurity scattering and increased secondary electrically inactive phase formation. There are numerous examples of the AACVD growth of cation doped ZnO using [Zn(OAc)_2_·2H_2_O] for TCO applications in the literature (Table 2). The electrical results from our study of P doped ZnO show that P is indeed a practically suitable dopant yielding enhanced n-type conductivity to levels acceptable for TCO application. In addition we compare our study with other P doped ZnO thin films from different precursors and synthesis methods (Table 3). The electrical properties were in a similar range (∼10^−3^ Ω cm) and the synthesis method in this study, AACVD, is considered as a more convenient method to prepare thin films with simple process and relatively low cost,^6,7,11^ which offers more advantages for TCO application.

**Table 2:** Compares with some other doped ZnO thin films through AACVD from the same Zn precursor^6,48–50^

Zn precursor	Dopant(s)	Dopant conc.	*ρ* (×10^−3^ Ω cm)	*n* (×10^20^ cm^−3^)	*μ* (cm^2^ V^−1^ s^−1^)	Ref.
[Zn(OAc)_2_·2H_2_O]	Sc	1.0 at%	1.2	7.2	7.5	6
[Zn(OAc)_2_·2H_2_O]	Cl	15 mol%	42.8	0.176	8.66	38
[Zn(OAc)_2_·2H_2_O]	Al, acetylacetone and DI water	2.9 at%	3.54	1.76	22.92	39
[Zn(OAc)_2_·2H_2_O]	In	3 at%	72	—	—	40
[Zn(OAc)_2_·2H_2_O]	P	6.5 at%	6.0	1.6	6.65	This study

**Table 3:** Compares with some other P doped ZnO thin films *via* different precursors and synthesis methods^41,51,52^

Zn precursor	P precursor	Dopant(s)	Synthesis method	*ρ* (×10^−3^ Ω cm)	*n* (×10^20^ cm^−3^)	*μ* (cm^2^ V^−1^ s^−1^)	Ref.
Diethylzinc	Trimethylphosphite	P	Atomic layer deposition	3	1.3	8.4	41
Purity ZnO	P_2_O_5_	P, O_2_	Pulsed laser deposition	10	—	—	33
Purity ZnO	P_2_O_5_	P, O_2_	RF magnetron sputtering	∼5	—	—	42
[Zn(OAc)_2_·2H_2_O]	[PO(OEt)_3_]	P	AACVD	6.0	1.6	6.65	This study

Ultraviolet-visible (UV-vis) spectra between 300 and 1100 nm for the undoped and P doped thin films on glass substrates are shown in the Fig. 3b. All films were transparent across the visible wavelengths (400–700 nm) with an average transmittance of ∼75%. The band gaps were determined by applying the Tauc formula to the UV/vis data and shown in Fig. 3c. The nominally undoped ZnO film had a band gap of 3.3 eV, which is close to previous literature Tauc plot results of 3.28 eV for undoped ZnO. Upon the introduction of P at 2.7, 3.4 and 6.5 at%, the ZnO band gap remains at 3.3 eV however at higher dopant concentrations of 8.6 and 14.3 at% the band gap widens to 3.5 eV.

## Conclusion

A series of undoped and P doped ZnO thin films with different concentrations of P were grown on glass substrates *via* AACVD. XPS showed P to be only in the V+ oxidation state on the surface. Evidence for successful doping and solid solution formation was provided by XRD analysis where only reflections for ZnO wurtzite phase were observed. Hall effect measurements showed that upon doping the electron density increased due to effective replacement of Zn^2+^ in the lattice with P^5+^. The lowest resistivity of 6.0 × 10^−3^ Ω cm was obtained at 6.5 at% P concentration and comparable to results obtained for ZnO:Al but better than ZnO:In from [Zn(OAc)_2_·2H_2_O] through AACVD. Optical measurements showed 75% transmittance to visible light and absorption in the NIR region for the films with high electron densities. The results of this study show that P doping of ZnO under a simple, saleable and industrially relevant technique such as AACVD with inexpensive and easy to handle precursors is able to produce sufficiently n-type conductive and stable thin films.

## Conflicts of interest

The authors declare no conflict of interest.

## Supplementary Material

RA-010-D0RA05667G-s001
