# Supplementary material for: n-Type conducting P doped ZnO thin films via chemical vapor deposition
Source: RSC Adv. 2020 Sep 17;10(57):34527–33. doi: 10.1039/d0ra05667g (PMC9056824; doi:10.1039/d0ra05667g)
Supplement: RA-010-D0RA05667G-s001 [file RA-010-D0RA05667G-s001.pdf]

Electronic Supplementary Material (ESI) for RSC Advances.
This journal is © The Royal Society of Chemistry 2020

n-Type Conducting P doped ZnO Thin Films via Chemical Vapor Deposition

Donglei Zhao, Jianwei Li, Sanjayan Sathasivam and Claire J. Carmalt*

*Corresponding author

Materials Chemistry Centre, Department of Chemistry, University College London, 20 Gordon Street, London WC1H 0AJ, UK

Fax: (+44) 20-7679-7463

E-mail: c.j.carmalt@ucl.ac.uk

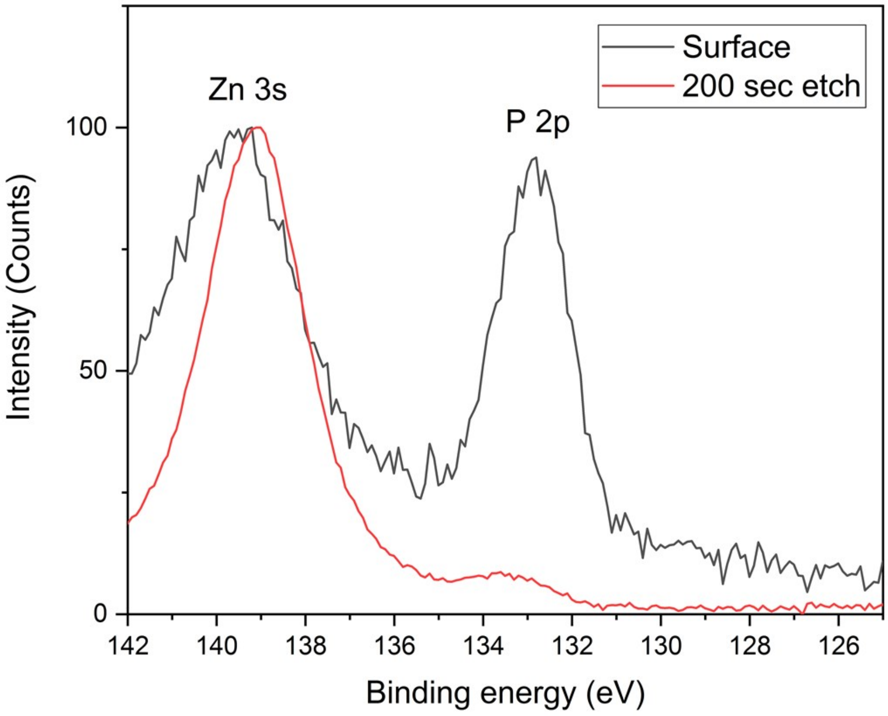

Figure S1: Depth profiling of the 14.3 at.% P doped ZnO films showing P to be highly surface segregated. XPS results show 52% P relative to Zn on the surface and only 2% after 200 seconds of etching
